# A survey of the sorghum transcriptome using single-molecule long reads

**DOI:** 10.1038/ncomms11706

**Published:** 2016-06-24

**Authors:** Salah E. Abdel-Ghany, Michael Hamilton, Jennifer L. Jacobi, Peter Ngam, Nicholas Devitt, Faye Schilkey, Asa Ben-Hur, Anireddy S. N. Reddy

**Affiliations:** 1 Department of Biology, Program in Molecular Plant Biology, Program in Cell and Molecular Biology, Colorado State University, Fort Collins, Colorado 80523, USA; 2 Department of Computer Science, Colorado State University, Fort Collins, Colorado 80523, USA; 3 National Center for Genome Resources, 2935 Rodeo Park Dr East, Santa Fe, New Mexico 87505, USA

## Abstract

Alternative splicing and alternative polyadenylation (APA) of pre-mRNAs greatly contribute to transcriptome diversity, coding capacity of a genome and gene regulatory mechanisms in eukaryotes. Second-generation sequencing technologies have been extensively used to analyse transcriptomes. However, a major limitation of short-read data is that it is difficult to accurately predict full-length splice isoforms. Here we sequenced the sorghum transcriptome using Pacific Biosciences single-molecule real-time long-read isoform sequencing and developed a pipeline called TAPIS (Transcriptome Analysis Pipeline for Isoform Sequencing) to identify full-length splice isoforms and APA sites. Our analysis reveals transcriptome-wide full-length isoforms at an unprecedented scale with over 11,000 novel splice isoforms. Additionally, we uncover APA of ∼11,000 expressed genes and more than 2,100 novel genes. These results greatly enhance sorghum gene annotations and aid in studying gene regulation in this important bioenergy crop. The TAPIS pipeline will serve as a useful tool to analyse Iso-Seq data from any organism.

## Abstract (web summary)

Alternative splicing and alternative polyadenylation (APA) contribute to mRNA diversity but are difficult to assess using short read RNA-seq data. Here, the authors use single molecule long-read isoform sequencing and develop a computational pipeline to identify full-length splice isoforms and APA sites in sorghum.

## Graphical abstract

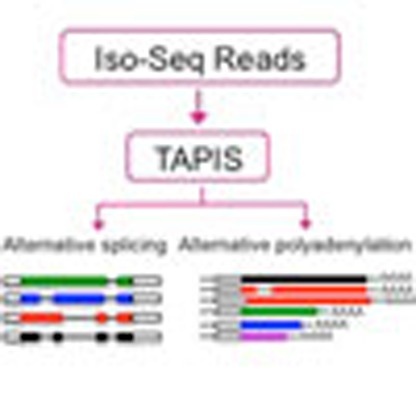

Recent high-throughput RNA sequencing (RNA-seq) studies using second-generation sequencing technologies have shown that eukaryotic transcriptomes are highly complex and that the post-transcriptional processing of precursor mRNAs including alternative splicing (AS) and alternative polyadenylation (APA) contribute significantly to enhance transcriptome diversity^1^,^2^,^3^,^4^,^5^. This transcriptome complexity plays an important role in determining the coding potential of genes and in regulating gene expression through multiple mechanisms^1^,^4^,^5^,^6^. Using short-read sequencing, it has been reported that ∼95% of intron-containing genes in humans and over 60% of multi-exon genes in plants are alternatively spliced^7^,^8^. Several studies in plants have shown that abiotic stresses can significantly impact AS of pre-mRNAs in plants^9^,^10^,^11^,^12^,^13^,^14^,^15^, and alternatively spliced genes have been shown to be over-represented in stress responses^16^,^17^,^18^. Interestingly, AS of plant pre-mRNAs encoding serine/arginine-rich proteins, which encode master regulators of both constitutive and AS, is dramatically altered in response to various abiotic stresses^11^,^15^,^19^. The changes in the levels of master regulators of splicing in response to stresses may alter the splicing of other pre-mRNAs including auto- and cross-regulation of splicing of serine/arginine-rich pre-mRNAs^9^,^10^,^12^,^13^,^14^,^20^. These studies suggest that altered ratios of splice variants in response to stresses may have a role in adaptation of plants to these stresses. Despite the fact that several large-scale RNA-seq studies have been performed in plants to analyse AS, currently it is not known how many distinct splice isoforms are produced^8^,^18^,^21^,^22^. This is primarily due to challenges associated with short-read sequencing in accurately reconstructing full-length splice variants. A recent study evaluated 25 transcript reconstruction protocols and found that although most methods achieve good precision and recall at the exon level (around 70% for human and up to 95% in *Caenorhabditis elegans*), accuracy was a lot lower for assembly of complete transcripts, even in *C. elegans* and *Drosophila melanogaster*, which have genes with much simpler transcript structure than humans^23^. This highlights the limitations in analysing short-read data with the available computational tools. Identification of full-length splice isoforms is necessary to deduce the nature of the encoded protein and in assessing a splice variant's role in gene regulation. Pacific Biosciences single-molecule long reads obtained using the Iso-Seq protocol offer a considerable advantage in transcriptome-wide identification of full-length splice isoforms and other forms of post-transcriptional regulatory events such as APA.

Recently, Iso-Seq has been used to analyse full-length splice isoforms in human organs and embryonic stem cells^24^,^25^. These studies have shown that even in a highly characterized transcriptome such as that of humans, identification of genes and splice isoforms is far from complete^24^,^25^. Thus far, the use of Iso-Seq for plants has been relatively limited^26^,^27^,^28^, particularly in relation to comprehensive analysis of splice isoforms and APA, and no established tools for the analysis of such data exist (except for software provided by Pacific Biosciences). Here we performed Iso-Seq with mRNA isolated from sorghum (*Sorghum bicolor* L. Moench) seedlings. Sorghum, a C_4_ crop plant used for food, feed, fibre and fuel, is one of the best-adapted cereals to drought and temperature; hence, used as a model system to investigate the molecular basis of adaptation to abiotic stresses^29^,^30^,^31^,^32^ (http://www.icrisat.org/crop-sorghum.htm). Furthermore, because of its ability to produce large amounts of biomass even under suboptimal conditions (for example, low inputs, reduced water and abiotic stresses), sorghum is also considered as an important bioenergy crop. Although the genome sequence of several sorghum lines has been completed recently^29^,^30^, the transcriptome is not well annotated; the extent of AS, the number of splice isoforms and transcriptome diversity due to APA are largely unknown. We applied Iso-Seq protocol to analyse the sorghum transcriptome and developed an easy to use pipeline called TAPIS to analyse Iso-Seq reads to identify full-length transcript isoforms and APA. The analysis of sorghum Iso-Seq data uncovered over 7,000 novel AS events, ∼11,000 novel splice isoforms, over 2,100 novel genes and several thousand transcripts that differ in 3′ untranslated regions due to APA. The results from sorghum Iso-Seq will permit reannotation of the sorghum transcriptome and serve as a valuable resource to the plant research community. Furthermore, the TAPIS pipeline would serve as a useful tool in analysing Iso-Seq data.

## Results

### Sorghum transcriptome sequencing using PacBio Iso-Seq

Short-read sequencing using the Illumina platform, the most widely used approach for RNA-seq, is powerful in quantifying gene expression and the detection of AS events. However, with the short-read data, it is challenging to accurately identify full-length splice variants generated from a gene^23^. To address this limitation, we sequenced the transcriptome of sorghum (BTx623) seedlings using the Pacific Biosciences Iso-Seq platform, which provides long reads—often up to transcript length, making it possible to accurately reconstruct full-length splice variants. RNA from control and drought stressed (polyethylene glycol (PEG)-treated) sorghum seedlings was used for Iso-Seq (see the Methods for details).

### Read mapping and error correction using TAPIS

Using the Iso-Seq protocol, we obtained 1,838,330 reads of insert (ROIs) of which 884,638 are full-length ROI (containing 5′ primer, 3′ primer and the poly(A) tail) and the rest are non-full-length ROI. The sizes of aligned ROI ranged from ∼20 to ∼3,886 nucleotides with an average read length of 1,042 (std=389) nucleotides (Supplementary Fig. 1). Although PacBio single-molecule sequencing yields long reads, it has a high error rate; in the Iso-Seq protocol, the error rate is lower since each position in a read is a consensus of several rounds of sequencing of the circular cDNA template. We measured the accuracy of the generated Iso-Seq reads against the published genome sequence assembly for sorghum BTx623 and observed a per-nucleotide error of 2.34% distributed as follows: mismatches (0.64%), insertions (1.07%) and deletions (0.63%). The average length for an insertion event was 1.23 nucleotides and 1.16 nucleotides for a deletion (Supplementary Fig. 2).

**Figure 1:**
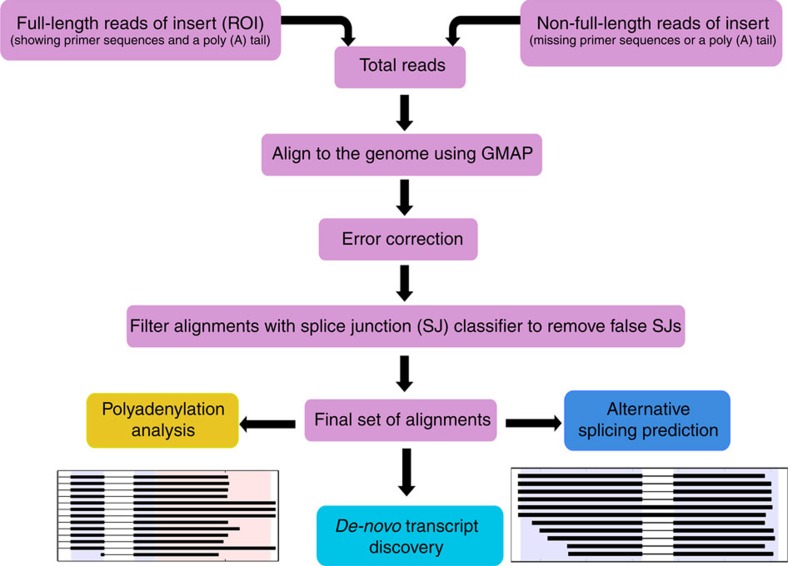
Transcriptome Analysis Pipeline for Isoform Sequencing. Schematic workflow of the transcriptome assembly and analysis pipeline for Pacific Biosciences Isoform Sequencing reads.

To analyse this data set, we developed TAPIS (Transcriptome Analysis Pipeline for Isoform Sequencing), a computational pipeline to correct errors, align reads to the reference genome, identify all splice isoforms and AS events generated from a gene and 3′ heterogeneity because of APA sites (Fig. 1). As the accuracy of read mapping is affected by read quality, especially for the correct identification of splice junctions, the first step in the TAPIS pipeline consists of an iterative process that alternates read mapping and error correction on the basis of the reference genome (TAPIS-ref; see the Methods for details). Using this approach, nearly 95% of the reads are aligned to the reference genome after filtering alignments for splice junction accuracy on the basis of a model of sorghum splice junctions. To demonstrate the effectiveness of TAPIS error correction, we compared its performance with an alternative that uses short-read Illumina data to perform read error correction with LoRDEC (Long-Read DBG Error Correction)^33^ or *proovread*^34^. Although using the LoRDEC- and proovread-corrected reads lead to high alignment rates, following filtering for splice junction accuracy, only 83% of the reads remain when using LoRDEC and 77% when using proovread (Table 1). This demonstrates the effectiveness of the TAPIS reference-guided approach that performs two-stage error-correction in the neighbourhood of splice junctions. A hybrid approach that first performs error correction using Illumina data followed by TAPIS reference-guided error correction led to a small improvement, with alignment rates of 96% using either LoRDEC or proovread. These results show that it is possible to achieve very high alignment rates of Iso-Seq reads without needing Illumina sequencing.

The distribution of Iso-Seq reads across sorghum chromosomes is shown in Supplementary Fig. 3. In pericentromic regions, which are known to be gene-poor and -rich in transposons^29^, especially long-terminal repeat retrotransposons, very little transcriptional activity was observed. Much of the transcriptional activity in all chromosomes is found on either side of pericentromic region that is gene rich (Supplementary Fig. 3).

From 867,089 reads with poly(A) sites, we identified 27,860 unique transcripts (see the Methods for details on how poly(A) sites were identified). Among these, 5,035 (18%) are intronless. Of the unique transcripts, 23,778 (85.3%) overlapped a single annotated protein-coding gene, 3,141 (11.3%) overlapped no annotated genes, which are likely novel genes (see Novel genes section below), and 941 (3.4%) overlapped two adjacent annotated genes, which either reflect misannotations in the current version of the sorghum annotations or represent read-through transcripts. In total, 14,550 genes have transcript coverage. For comparison, we find that using close to 110 million RNA-seq Illumina reads from the same biological samples around 17,750 genes had an fragments per kilobase of transcript per million mapped reads (FPKM)>2.2, which is the coverage threshold value used in a recent paper^35^ to obtain reliable assemblies using Illumina data. This demonstrates that we have transcript predictions for ∼82% of the expressed genes for which reliable transcript assemblies using Illumina data can be made. A small percentage of reads (<4%) that aligned to more than one location were separated. Further analysis has shown that some of these reads aligned to long-terminal repeat transposons in the pericentromic region of chromosomes.

### Splice isoforms and AS

**Figure 2:**
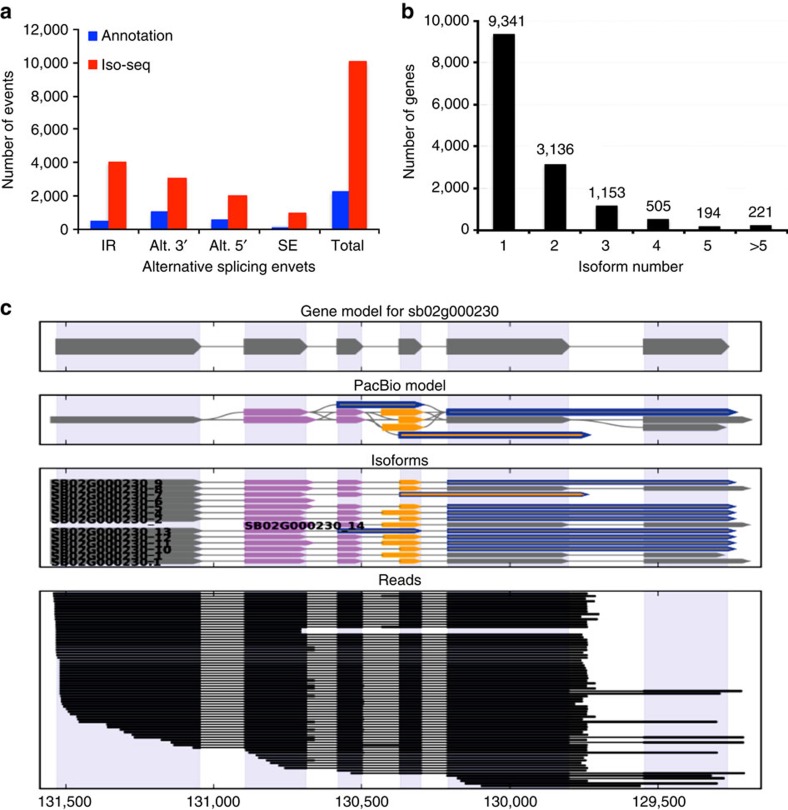
Alternative splicing and splice isoform analysis with Iso-Seq reads. (**a**) The total number of AS events in genes expressed in seedlings based on Iso-Seq data compared with the annotated gene models. Annotation, AS events in genes expressed in seedlings based on gene models; Iso-Seq, AS events in genes expressed in seedlings based on Iso-Seq reads. Alt 3′, alternative 3′ splicing; Alt 5′, alternative 5′ splicing; ES, Exon skipping; IR, intron retention; Total, All AS events. (**b**) Distribution of genes that produce one or more splice isoforms in seedlings. (**c**) An example of a gene that produces 13 novel splice isoforms. The gene models contain a single splice isoform for this gene. Gene model (top), splice graph (middle) and aligned reads (bottom) are shown.

About 80% of protein-coding genes and many non-coding RNAs (for example, microRNAs (miRNAs), long non-coding RNAs and others) in plants contain introns that are excised to generate mature mRNAs^4^. Previously, it was reported that pre-mRNAs of ∼1,500 genes undergo AS in sorghum^29^. In this work, we demonstrate that this number is much higher. Figure 2a shows the statistics of AS events detected in sorghum based on Iso-Seq reads and corresponding annotated gene models. We detected a total 10,053 AS events from the Iso-Seq reads, whereas only 2,950 events are present in the gene models (Fig. 2a). Using expressed sequence tags (ESTs), another study reported only 2,137 AS events^36^. Identification of over 7,000 new AS events in our study suggests that many AS events are not annotated in the published version of the sorghum genome^2^. The distribution of AS events is similar to other plants, with the majority of AS events being intron retention events.

Currently, it is not known how many full-length splice isoforms are produced in sorghum. In our Iso-Seq analysis, we detected a total of 27,860 transcripts, of which 11,342 (40.7%) are novel. Furthermore, 7,065 (25.4%) were identified as full-length, that is, the starting exon of an isoform aligned with the first exon of an annotated gene. Figure 2b summarizes the isoform numbers from Iso-Seq reads. In 9,341 genes (69.9%), only a single isoform was detected. About 5,200 genes produced two or more transcripts for a total of 14,437 isoforms (Fig. 2b). For 415 genes, 5 or more splice isoforms were detected. Overall, Iso-Seq revealed more than 11,300 novel splice isoforms in this study. An example of a gene that is annotated to produce a single transcript but is found to generate 14 splice variants is shown in Fig. 2c. Splice isoforms detected from all expressed genes are made available on our website (http://combi.cs.colostate.edu/sorghum/isoseq/).

**Figure 3:**
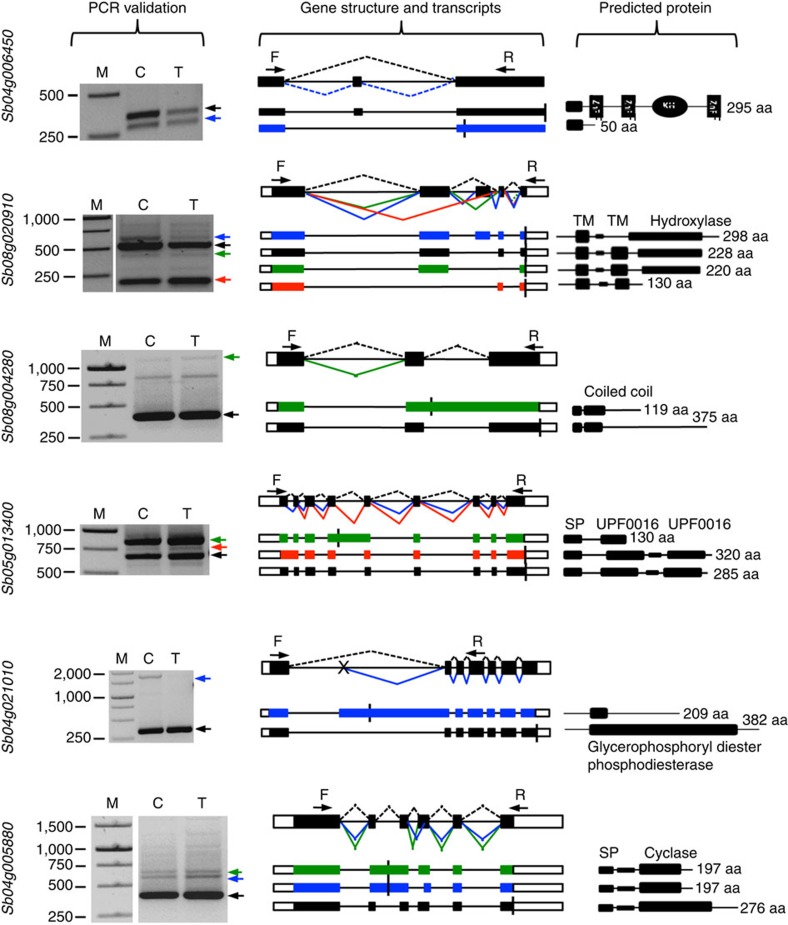
PCR validation of alternative splicing events identified by Iso-Seq. cDNA from control (C) and treated (T) samples was used for PCR. Primer sets (F, forward and R, reverse) were designed to flank the splicing events. PCR products were excised from the gel, purified, cloned into pGEM-T Easy Vectors and sequenced from both directions. Sequences were aligned to the corresponding gene sequence and the structures of the novel isoforms were verified. Exons are represented by filled boxes, introns by lines and 3′ and 5′ UTRs are represented by open boxes. The gene models have one annotated isoform, which is shown in black. The novel isoforms that are supported by PacBio reads and/or sequencing of PCR products are colour-coded and indicated by arrows. Different splicing events are represented by lines connecting exons. Predicted protein for each isoform and putative domains predicted using the simple modular architecture tool (SMART) are presented in the right panel. The location of the predicted stop codon in the transcripts is represented by a vertical line. Alt. 3′, alternative acceptor site; Alt. 5′, alternative donor site; ES, exon skipping; IR, intron retention; SP, signal peptide; TM, transmembrane; M, lane with DNA size markers. Gene ID is shown at the left for each panel.

To validate the accuracy of the splice isoforms detected with Iso-Seq reads, we randomly selected six genes that are annotated to produce a single transcript, but showed two or more isoforms based on Iso-Seq reads. We designed primers for the first and last exons or appropriate regions of these genes (Supplementary Table 1) and performed reverse transcription (RT)–PCR using RNA from control and PEG-treated seedlings. The gel banding pattern and the size of the fragments were consistent with the splice isoforms identified from Iso-Seq data (Fig. 3). We then cloned each of the predicted size fragments based on Iso-Seq and sequence-verified splice junctions and splice isoforms. The sequences of the verified isoforms and their alignment against the corresponding genes are shown in Supplementary Data 1. Information on clones that provided evidence in support of each isoform is presented in Supplementary Fig. 4. All types of AS events (exon skipping, intron retention and alternative 5′ and 3′ splicing) are represented in these verified isoforms. All isoforms that have Iso-Seq evidence were confirmed by sequencing. We also found that some splice isoforms are differentially expressed in response to drought stress. For instance, one of the transcripts from *Sb04g021010* is presented only in control seedlings and also the ratio of two splice isoforms from *Sb04g006450* is different between control and treated seedlings (Fig. 3).

### Cufflinks analysis

To demonstrate the advantage of splice isoform identification using PacBio reads, we compared the TAPIS assembly with a short-read assembly generated using Cufflinks using close to 110 million Illumina reads obtained from the same biological samples as the PacBio reads. We found that although 93.1% of the PacBio splice junctions were found in the short-read assembly, only 59% of the splice isoforms were recovered, suggesting that Cufflinks identifies less than two-thirds of all splice isoforms.

### Alternative polyadenylation

Polyadenylation of the 3′ end of most mRNAs, an important co-transcriptional modification in most eukaryotic transcripts, is necessary for transport of RNA to the cytoplasm, and its localization, stability and translation^5^,^37^. Transcripts derived from a given gene have been shown to contain different 3′ ends because of alternative cleavage and polyadenylation^3^,^5^,^38^,^39^. Recent high-throughput studies have revealed that APA enhances transcriptome complexity by generating transcript isoforms that differ in the coding region or 3′ untranslated regions, thereby regulating gene expression through multiple mechanisms in both plants and animals^2^,^3^,^5^,^39^. Differential polyadenylation of mRNAs has been shown to play an important regulatory role in plant development especially flowering^40^,^41^. Although identification of 3′ ends of transcripts from a gene is critical, it is not possible to identify APA with short-read data obtained with the conventional RNA-seq approach. In *Arabidopsis*, a specialized poly(A) tag sequence or direct RNA sequencing was used to identify transcript isoform differences due to APA^2^,^3^.

In sorghum, the transcriptome complexity due to APA is unknown. As our Iso-Seq libraries were made using an oligo dT primer for cDNA synthesis, the 3′ ends of all transcripts are represented in our reads. The TAPIS pipeline includes utilities for analysing the heterogeneity in 3′ end formation, and yielded the following results. Of the 14,550 expressed genes, 11,013 have at least one supported poly(A) site (see the Methods section). The average number of poly(A) reads aligning to an annotated gene is ∼55, resulting in 38,736 unique poly(A) sites. A total of 2,301 (20.9%) genes having at least one supported poly(A) site have annotated start and stop codons. From these genes, location distributions from 5,641 sites were calculated. As expected, most (5,437 (96.4%)) poly(A) cleavage sites were detected in the 3′ untranslated region (UTR) region. However, a number of internal cleavage sites (200 (3.5%)) were found along with a few (4 (0.1%)) in the 5′ UTR. These values are similar to the published distributions of poly(A) in *Arabidopsis*^2^,^3^. Furthermore, for 2,301 genes that have annotated 3′ UTRs in the gene models, our data revealed novel 3′ ends due to APA. The distribution of cleavage site locations and the existence of the poly(A) signal upstream of cleavage site (see below) indicate that we are able to accurately annotate poly(A) sites and APA in sorghum with Iso-Seq reads. To assess how many of the poly(A) sites in sorghum ESTs are represented in our analysis, we compared our results with the available EST data. A total of 1,601 genes were found in both the Iso-Seq data and EST data that have poly(A) site evidence. From 2,179 poly(A) sites detected in ESTs, 1,554 (71.3%) were supported by our Iso-Seq analysis (see Supplementary Fig. 5 for details), suggesting that our method accurately identifies bonafide poly(A) sites.

**Figure 4:**
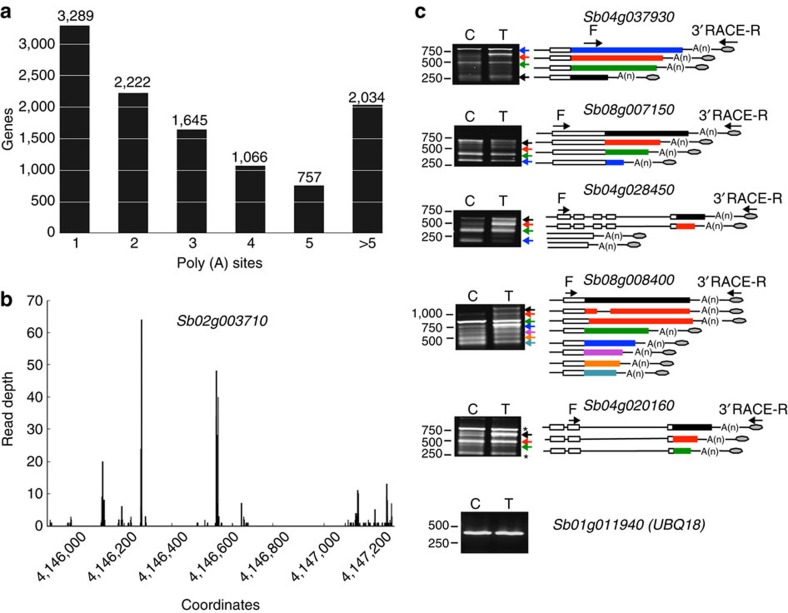
Alternative polyadenylation analysis. (**a**) Distribution of the number of poly(A) sites per gene. Poly(A) reads were clustered such that each site must have at least 2 reads supporting it and no two clusters are within 15 nucleotides of each other. (**b**) An example of a gene that produces transcripts with multiple polyadenylation sites in 3′UTR. Distribution of number of poly(A) reads (*Y* axis) along with estimated cluster centres shown as vertical lines on the *x* axis. (**c**) Validation of polyadenylation sites by PCR. cDNA was prepared from RNA extracted from control (C) and treated (T) tissues using 3′ RACE adaptor primer and PCR was carried out using 3′ RACE reverse (R) primer and gene-specific forward (F) primer. Sizes of PCR products were calculated and compared with the predicted products calculated from Iso-Seq. Exons are represented by open boxes and 3′ UTRs were represented by coloured boxes. Equal amount of cDNA in samples is verified using UBQ as an internal control.

As microheterogeneity in poly(A) is common in plants^2^,^3^,^38^, poly(A) sites in transcripts from a gene that are present within 15 nucleotides of each other were clustered together and considered as part of a single polyadenylation site^3^. Using this criterion, transcripts from over 7,700 genes contain two or more polyadenylation sites (Fig. 4a, Supplementary Data 2, http://combi.cs.colostate.edu/sorghum/isoseq/), suggesting that APA is a common phenomenon in sorghum. Of these, around 3% were in the coding region or the 5′ UTR. We checked if there is a genome-encoded stretch of ‘A' downstream of cleavage sites that occur in the coding region and detected no enrichment of ‘A' downstream of cleavage sites in the genomic region, suggesting that these are not due to oligo dT internal priming. Among those that have multiple alternative poly(A) sites, 60.1% have a preferred site—a poly(A) site containing more than 50% of the poly(A) reads aligning to a poly(A) site. Figure 4b shows an example of a transcript with multiple polyadenylation sites with the supporting Iso-Seq read depth for each poly(A) site. We validated all the APA events in several randomly selected genes using 3′ rapid amplification of cDNA ends (3′RACE; Fig. 4c). The number and size of the fragments confirmed that these are authentic 3′ ends. A recent study using poly(A)-tail length sequencing (PAL-Seq) showed that the median length of poly(A) tail in *Arabidopsis* is 51 nucleotides^42^, hence different sizes of transcripts observed are not likely due to poly(A) tail length. For only one gene, additional APA events (*Sb04g020160*) that were not found in Iso-Seq reads were detected. In addition, we have observed differential APA because of drought treatment (Fig. 4c, see bands indicated with red, black and blue arrows in the PCR panel of *Sb04g028450* and red and black arrows in the PCR panel for *Sb08g008400*). This indicates that APA is a regulated phenomenon.

The median length size of 3′ UTRs in sorghum is 280 nucleotides. With the direct RNA sequencing of poly(A) tags^2^, it was not possible to determine the length distribution of 3′ UTRs as it yielded only short reads. It has been shown that variation in the length of 3′ UTRs has a role in miRNA-dependent regulation of gene expression by the presence or absence of a site for an miRNA^43^,^44^. Therefore, we searched for miRNA targets in transcripts with different 3′ ends due to APA or AS. Only for a few genes, we detected an miRNA target sequence in one transcript isoform and not the other, suggesting that these transcripts may be subjected to miRNA regulation. One of the features that determines the stability of mRNA is the length of 3′ UTR^44^. Transcript isoform diversity due to APA also increases or decreases 3′ UTR length. The median difference in 3′ UTRs due to APA is 60 nucleotides, suggesting that the variations in 3′ UTR may be involved in mRNA stability.

**Figure 5:**
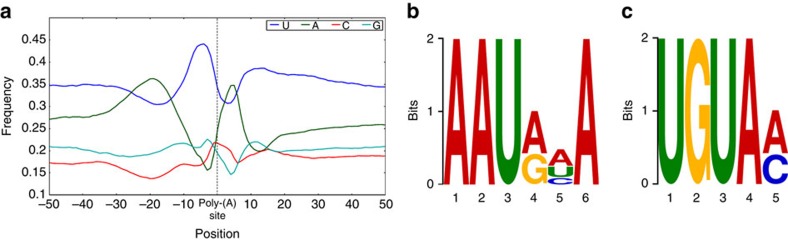
Analysis of sequence elements at cleavage sites. (**a**) Nucleotide composition around poly(A) cleavage sites. The relative frequency of a nucleotide is shown as a function of genomic position across all poly(A) cleavage sites detected in our data. The low GC-content and poly(A) spike after the cleavage site is in agreement with the poly(A) analysis reported for *Arabidopsis*^3^. (**b**) MEME analysis identified a poly(A) signal in sorghum transcripts. An over-represented motif at 25 nts upstream of the poly(A) site similar to the known signal in dicots was identified. (**c**) Another overrepresented motif (UGUA) is found about 35 nts upstream of the poly(A) site.

We analysed the nucleotide composition in the upstream (−50 nts) and downstream (+50 nts) of all poly(A) cleavage sites for nucleotide bias. There was clear bias of nucleotides upstream and downstream of the cleavage site in 3′ UTRs (see Fig. 5a for details). The nucleotide bias in these sites is similar to 3′ UTR profiles reported earlier^2^,^3^, suggesting that the identified poly(A) sites are authentic sites. To identify potential *cis*-elements necessary for polyadenylation, we performed a MEME analysis^45^ for motifs enriched upstream of the cleavage site using 50 nucleotides upstream from the predominant poly(A) site of all transcripts. This analysis identified a significantly over-represented polyadenylation signal (AAUAAA), a known canonical poly(A) signal in both plants and animals, 25 nts upstream of the cleavage site (Fig. 5b)^5^,^46^,^47^. In addition, a UGUA motif was found around 35 nts upstream of the poly(A) site (Fig. 5c). This conserved motif in eukaryotes is known to bind cleavage factor IIm^5^,^47^

### Differential gene expression analysis

Identification of differentially expressed genes is one of the primary analyses performed using Illumina sequencing data. Although our Iso-Seq data have roughly 20 times less reads than a typical RNA-seq experiment, the length of the reads compensates for the low number of reads to some extent. We used GFOLD, which is specifically designed for data without biological replicates to search for differentially expressed genes between the control and treated samples^48^. Our analysis yielded 186 differentially expressed genes with a threefold cutoff (85 upregulated in the treated sample and 101 downregulated; Supplementary Data 3). We experimentally confirmed differential expression of ten randomly chosen genes using RT–qPCR (see Supplementary Fig. 6 for details).

### Novel genes

**Figure 6:**
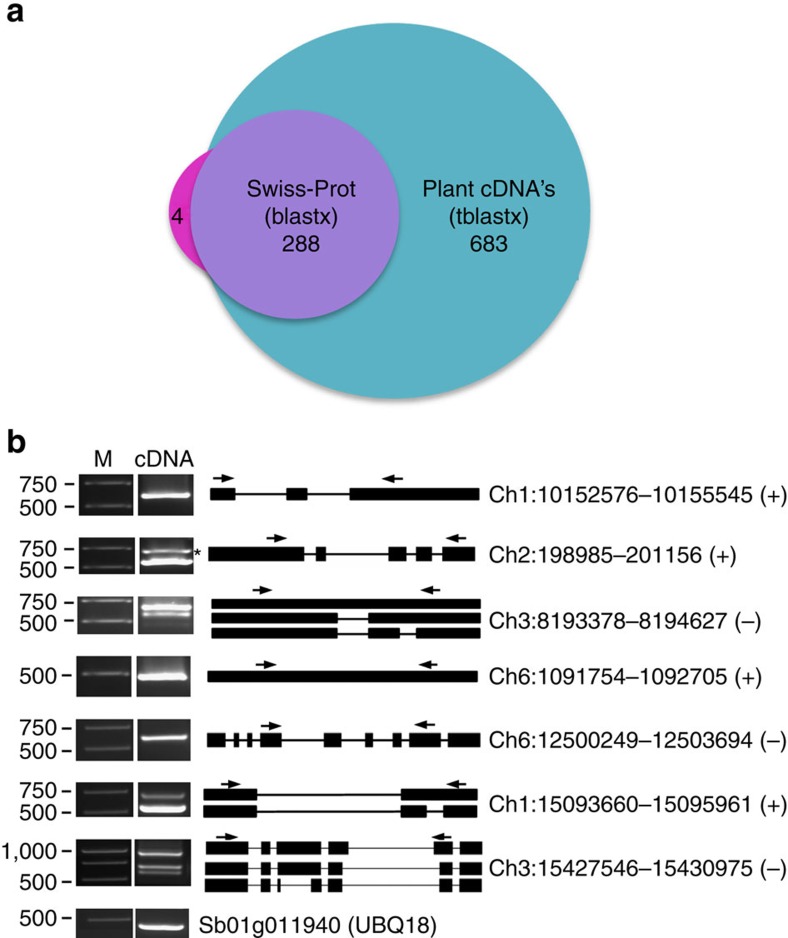
Novel genes identified in Iso-Seq data. (**a**) The number of novel genes in sorghum that showed significant sequence similarity in a blastx search against Swiss-Prot proteins or a significant match in a tblastx search against plant cDNAs. (**b**) RT–PCR validation of novel genes and putative long non-coding RNAs. Single exon genes, genes with multiple exons and predicted long non-coding RNAs were validated by PCR using cDNA prepared from 8-day-old seedlings. Forward and reverse primers are shown as arrows. The schematic representation of genes is shown. Exons are represented as black boxes and introns as lines. The coordinates of the genes on the chromosomes were shown at the right. Plus and minus signs represent the forward and reverse strands, respectively.

The published version of sorghum genome annotations contains ∼34,500 gene models, with transcript or homology-based evidence for over 27,000 of them^29^. Among the unique transcript clusters found in our analysis, 2,171 did not overlap any annotated gene, hence are likely novel genes (for coordinates see http://combi.cs.colostate.edu/sorghum/isoseq/). To assess the presence of these novel genes in other organisms, we conducted two BLAST analyses using tblastx and blastx as described in the Methods section. In total, we detected 971 (44.7%) clusters with at least one significant match in the tblastx search against a collection of plant cDNAs from protein-coding genes (Supplementary Data 4) and 288 (∼13%) were found in the blastx search against Swiss-Prot proteins (Fig. 6a). Of the 288 clusters found using blastx, all but 4 have significant hits in the tblastx search (Fig. 6a). Given this high overlap, we believe the 971 clusters detected in the cDNA experiment likely represent transcripts from novel protein-coding genes in sorghum. Among the novel genes, we found single exon and multi-exon genes and some of the multi-exon novel genes exhibited AS. To verify the expression of some of the novel genes, we randomly selected seven genes (with or without exons and with or without AS) and performed RT–PCR. As shown in Fig. 6b, expression and splicing were confirmed in our expression analysis.

### Non-coding RNAs

Less than 1% of the reads aligned to non-coding sequences, which include miRNAs and long non-coding RNAs. A total of 149 miRNAs were annotated in sorghum^29^. It was proposed that recent gene and miRNA duplications may have contributed to drought tolerance^29^. For read clusters that did not overlap an annotated gene, we performed a BLASTN search against miRNA stem-loop sequences (28,645 records) curated at miRBase^49^,^50^,^51^,^52^. Specifically, we used the longest read in a cluster as the ‘cluster representative' and used an *e*-value threshold of 10^−5^ to determine if the representative read was a significant hit. In all, 49 read clusters were identified from 9 chromosomes and 6 scaffolds. Of these 49 clusters, 20 match miRNA only from other species, suggesting these are novel sorghum miRNAs (Supplementary Data 5). Finally, 14 miRNA clusters have evidence for introns and 11 of these exhibited AS (Supplementary Data 5). We verified AS of three miRNAs (miR159, miR166 and miR444) using RT–PCR (Supplementary Fig. 7, see Supplementary Data 1 for sequences of splice variants). Furthermore, the relative abundance of the splice isoforms from all three is altered in drought stressed seedlings. 540 genomic locations with Iso-Seq expression exhibit properties of lncRNA: the longest representative transcript of these regions have no ORFs whose length is greater than 200 nucleotides^53^,^54^. Our analysis with Iso-Seq reads has also revealed 763 annotated genes where a single TAPIS transcript assembly overlapped two or more sorghum genes. These genes are likely fragments of a complete gene. In addition, we found 178 annotated genes that overlap two or more TAPIS transcript assemblies, suggesting that these annotations are an entanglement of multiple genes.

## Discussion

Our results provide the first comprehensive view of splice variants in sorghum, underscoring the advantage of Iso-Seq in identifying full-length splice isoforms. In summary, our sorghum Iso-Seq data yielded a number of novel findings including: (i) identification of transcriptome-wide full-length isoforms in sorghum at an unprecedented scale with over 11,000 novel splice isoforms, (ii) detection of ∼11,000 new splicing events from the Iso-Seq reads as compared with splicing events in the gene models, (iii) uncovering of extensive APA of sorghum transcripts (∼50% of expressed genes were found to have multiple polyadenylation sites) and (iv) identification of over 2,100 novel genes that were not previously annotated. Among the novel genes, many are putative long non-coding transcripts. Our results show the potential of Iso-Seq for characterizing transcriptome-wide splice variants and APA in plants, and our predicted splice isoforms and APA sites would be a great resource in reannotating the sorghum genome; furthermore, the effectiveness of Iso-Seq for detecting APA eliminates the need for a separate protocol for this task. Similar analysis in other plants will greatly contribute to identifying full-length isoforms and understanding the role of AS and APA in gene regulation and the evolutionary conservation of full-length splice forms. Our easy to use computational pipeline would be a valuable resource to the broader scientific community in analysing Iso-Seq data. Furthermore, this pipeline can also be used in the absence of Illumina short-read data as we have shown that error correction using just the reference genome is almost as good as using both. Sequencing of the sorghum transcriptome with RNA from different tissues and under different conditions in the future will likely lead to further discovery of novel splice variants and APA, identification of tissue- and development-specific full-length splice isoforms, and help in further refining the annotation of the sorghum genome.

Our validation studies confirmed the splice isoforms and differential polyadenylation sites detected in this study for a number of genes. Although this study resulted in the identification of a large number of full-length splice isoforms and novel genes, further refinements in library preparation such as enrichment of full-length transcripts using 5′ cap capture following poly(A) RNA purification and reducing or eliminating PCR amplification pre- and post-fractionation of cDNA might uncover even more full-length splice isoforms, APA events and low abundance splice isoforms. As stresses appear to have a dramatic effect on post-transcriptional events, especially AS, performing Iso-Seq with RNA form plants that are subjected to various stresses will allow for the identification of stress-regulated isoforms globally and also uncover the impact of stresses on APA. Such information would be vital in addressing the role of stress-regulated post-transcriptional splicing and polyadenylation in plants' adaptation to diverse stresses.

## Methods

### Plant materials and growth conditions

Seeds of the *Sorghum bicolor BTx623* genotype were surface sterilized in 20% bleach for 30 min, rinsed five times with sterile distilled water and then allowed to germinate on a wet filter paper for 24 h. Germinated seedlings were then transferred to culture tubes and allowed to grow vertically on a × 0.5 Hoagland's solution-wetted 3 M filter paper bridge for an additional 7 days under 16 h light/8 h dark, 50% humidity and 26 °C day/night temperature. On the 8th day, the media were completely decanted and replaced with 5 ml of × 0.5 Hoagland solution (control) or 5 ml of 20% PEG-8000 (Sigma) dissolved in × 0.5 Hoagland solution (treated). Tubes were incubated for 6 h under continuous light in the same growth chamber. Seedlings, excluding the remnants of the seeds, were harvested, rinsed with cold distilled water and frozen in liquid nitrogen.

### PacBio Iso-Seq library preparation and sequencing

The Iso-Seq library was prepared according to the Isoform Sequencing protocol (Iso-Seq) using the Clontech SMARTer PCR cDNA Synthesis Kit and the BluePippin Size Selection System protocol as described by Pacific Biosciences (P/N100-377-100-05 and P/N100-377-100-04) with the following modifications. For cDNA conversion, 3 μg of total RNA was used as input into the Clontech SMARTer reaction. A total of 23 PCR cycles of amplification was performed using Phusion DNA polymerase (NEB, Cat: M0530L). Amplification was followed by size selection using the BluePippin (Sage Science) of the following bins for each sample: 1–2 and 2–6 kb. After size selection another amplification was performed using 12 PCR cycles. The amplified and size selected cDNA products were made into SMRTbell Template libraries according to the Iso-Seq protocol referenced above. Libraries were prepared for sequencing by annealing a sequencing primer (component of the SMRTbell Template Prep Kit 1.0) and binding polymerase to the primer annealed template. The polymerase-bound template was bound to MagBeads (P/N 100-125-900) (https://goo.gl/wdZErU) and sequencing was performed on a PacBio RS II instrument. Fourteen v3 SMRTcells (Pacific Biosciences, P/N 100-171-800) were run for each sample for a total of 28 SMRTcells. The libraries composed of transcripts from the 1–2 kb range were each sequenced on two cells using P4C2 polymerase and chemistry (currently discontinued) and 180 min movie times and three cells using P6C4 polymerase (Pacific Biosciences, P/N 100-372-700) and chemistry (Pacific Biosciences, P/N 100-356-200) and 240 min movie times. The libraries composed of transcripts from the 2–6 kb range were each sequenced on nine cells with two cells using P4C2 polymerase and chemistry and 180 min movie times, and seven cells using P6C4 polymerase (Pacific Biosciences, P/N 100-372-700) and chemistry (Pacific Biosciences, P/N 100-356-200) and 240 min movie times.

Each size fraction for each sample was run through the Iso-Seq pipeline included in the SMRT-Analysis software package individually. First, ROIs (previously known as circular consensus sequence) were generated using the minimum filtering requirement of 0 or greater passes of the insert and a minimum read quality of 75. This allows for the highest yield going into the subsequent steps, while creating higher accuracy consensus sequences where possible. The pipeline then classified the ROI in terms of full-length nonchimeric and non-full length reads. This is done by identifying the 5′ and 3′ adapters used in the library preparation as well as the poly(A) tail. Only reads that contain all three in the expected arrangement and do not contain any additional copies of the adapter sequence within the DNA fragment are classified as full-length non-chimeric (https://github.com/PacificBiosciences/cDNA_primer/wiki/Understanding-PacBio-transcriptome-data).

### Transcriptome analysis pipeline for isoform sequencing

The frequency of nucleotide indels and mismatches are much higher in PacBio Iso-Seq reads than in shorter high-throughput sequencing and can lead to incorrectly detected splice sites. A common approach to correct these errors is to align short reads to long reads using programmes such as ‘proovread'^34^. Error correction in our TAPIS pipeline is performed using an iterative alignment-correction approach that uses the reference genome. Full -length and non-full-length reads are first aligned to the reference genome (and any available splice junction annotations) using the Genome Mapping and Alignment Program (GMAP)^55^ with the options: --no-chimeras, --cross-species and setting k (the maximum intronic length) to the length of the largest intron found in the gene annotations of that species. Next, indels and mismatches are corrected using the reference genome. In the first iteration, errors in reads near splice junctions are left uncorrected to improve accurate detection of splice sites in subsequent rounds of alignment.

LoRDEC and proovread read correction were conducted using an RNA-seq data set consisting of 419,760,833 reads from 3 stress treatments and a control collected from root and shoot tissues^56^. Following alignment and error correction, reads were screened through two filtering criteria. First, reads that could not be assigned to a strand were eliminated. These reads include those that did not have an identifiable poly(A) tail, and reads aligning to the opposite strand predicted from the SMRT DNA Sequencing platform (v2.2) from Pacific Biosciences (http://www.pacb.com/devnet/). Finally, splice junctions from gapped alignments were filtered using *SpliceGrapher*^57^ to remove false positives. Alignment and filtering results are summarized in Table 1. The total number of reads and percentage with respect to the total number of reads are shown for each library. The order of filtering is as follows: Aligned Reads→Stranded Reads→Splice Junction Filtered Reads.

Filtered reads were then assembled into clusters such that reads of each cluster overlapped another read. Clusters were then processed into unique splice isoforms by merging reads that have the same intronic coordinates; we also merge reads whenever all the intron coordinates on their 3′ end are identical. Because of the occurrence of APA, we did not consider reads that differ only in the downstream coordinates of 3′-most exons as unique splice isoforms. From the reads that represent a splice-isoform, we construct a representative transcript by using the read that includes the largest number of exons and extending it to the 3′-most poly(A) site, and extending its first exon to the 5′-most coordinate represented in the collection of reads that support the isoform.

Unique isoforms processed from clusters are then associated with genes using annotation provided in either GFF or GTF format. Isoforms spanning two or more genes are removed from downstream splice isoform analysis; however, they likely represent misannotations in the gene models. Isoforms not associated with a gene are considered novel. Furthermore, isoforms with 5′ exons occurring within a first exon of an annotated isoform are considered full-length.

AS analysis was conducted using *SpliceGrapher*^57^ by converting detected splice isoforms into splice graphs. Introns fully subsumed by an exon were labelled as retained. Overlapping exons that differed at their 5′ or 3′ splice junctions were considered as alternative 5′ or 3′ splicing events, respectively. Finally, exons absent in other isoforms were considered as exon skipping events.

Analysis of poly(A) is carried out using all reads aligning to each annotated gene. To address the possibility of small alignment artefacts at the 3′ end of reads and to ignore microheterogeneity of APA, reads were processed to form unique poly(A) sites. We adopted a greedy strategy by computing the depth of each candidate poly(A) site as the number of reads aligning within a window of five nucleotides of the site. The site with maximum read depth across all candidates is added to the list of poly(A) sites if the site has depth of at least two and does not occur within 15 nucleotides of a previously added site^3^. The site is then removed from the list of candidates and the algorithm proceeds until no candidate sites remain.

### Sorghum poly(A) analysis

Poly(A) sites from genes with annotated START and STOP codons were classified into 5′ UTR, 3′ UTR or internal if the position of the site occurred upstream of the START codon, downstream of the STOP codon or between the START and STOP codon, respectively. In cases where multiple START or STOP codons were present, we compared the sites to codon coordinates that minimize the size of the UTRs.

To computationally verify our detected poly(A) sites, 209,835 sorghum EST sequences were downloaded from NCBI using the following search URL: http://www.ncbi.nlm.nih.gov/nucest/?term=%22Sorghum+bicolor%22%5Bporgn%3A__txid4558%5D (last accessed 11 June 2015, 18:30 UTC-6) and subsequently aligned to the sorghum reference genome using GMAP with the following options: --no-chimeras --cross-species --expand-offsets 1 -B 5 -K 8000 -f samse -n 1 -t 20. Aligned reads were then screened for poly(A) evidence by selecting those that aligned with soft trimming at the end of the read and whose sequence terminates with a stretch of A nucleotides the same length of the trimming. Similarly, for reads aligned in reverse orientation, we looked for alignments with soft trimming at the beginning of the alignment with a corresponding stretch of T's at the beginning of the sequence. Comparison with poly(A) sites found using our pipeline were carried out using only genes found in both data sets.

To identify poly(A) sequence signals in our data, we performed a MEME analysis on the sequence of 50 nucleotides upstream of the 11,013 poly(A) sites. MEME-ChIP was run locally on a Linux system with the following options: meme-chip -norc -rna -oc primary_memechip -meme-minw 6 -meme-maxw 6 -meme-p 4 primary50.fa -bfile background.txt, where background.txt is a first-order Markov probability file built using the fasta-get-markov script from the MEME suite over all input sequences.

### Novel genes

Read clusters, as defined above in the TAPIS method section, that overlap no annotated genes were classified as novel genes. The coding potential of read clusters was assessed using both blastx and tblastx as described in the next section.

### BLAST analyses

In each BLAST run, we selected the longest read in a cluster as the representative sequence for searching the databases. For the tblastx analysis, we built a database from all plant cDNAs of protein-coding genes from Ensembl Plants, release 27 (http://plants.ensembl.org/info/website/ftp/index.html, last accessed on 2 July 2015). We then removed records for proteins described as hypothetical, predicted or non-coding, resulting in a database of 362,599 sequences. For blastx, we used the Swiss-Prot database, release 2015_07 (ftp://ftp.uniprot.org/pub/databases/uniprot/current_release/knowledgebase/complete/uniprot_sprot.fasta.gz, last accessed on 2 July 2015) containing 548,872 protein sequences. Finally, we ran tblastx and blastx with the options ‘-strand plus' and ‘-evalue 10e-5'.

### Non-coding RNA

Putative miRNA transcripts were identified using a BLAST analysis using all plant miRNAs from miRBase—a repository of miRNAs from several species. Novel genes not identified as having coding potential or exhibiting miRNA homology were analysed for lncRNA potential by identifying novel genes where the longest representative transcript has no ORF>100 amino acids yet has a nucleotide sequence of at least 350.

### Cufflinks analysis

Single-end, length-88 RNA-seq libraries from our treated and control sorghum samples were used (56,672,860 from the control and 53,085,767 from the PEG-treated samples). The reads were aligned using TopHat with the command line arguments -p 8 -i 10 -I 10,000 --b2-sensitive --segment-length 20 –GTF. The resulting alignments were provided to Cufflinks using the following options: -g genome_v1.gtf -p 4 -m 88 -s 1 -I 8,000 --library-type ff-firststrand, yielding 50,398 gene records. The isoform-level recall was computed as the percentage of TAPIS-assembled isoforms that are represented in the cufflinks assembly. For the splice junction recall, the percentage of all splice junctions from the TAPIS-assembly found in the cufflinks assembly was reported.

### RNA extraction and RT–PCR

Total RNA was extracted using the TRIzol reagent (Invitrogen) as described in the user's manual and its integrity was checked using Bioanalyzer (Agilent Technologies). For cDNA synthesis, 2.5 μg of total RNA was treated with DNase I (Fermentas) and then used to synthesize the first-strand cDNA using SuperScript III (Life Technologies) primed with oligo dT. For PCR validation of AS and novel transcripts, 1 μl of the diluted cDNA (1:5) was used in a reaction volume of 20 μl using TaKaRa Ex Taq DNA Polymerase (Clontech). Gene-specific primers were designed to span the predicted splicing events using Primer3Plus software (http://www.bioinformatics.nl/cgi-bin/primer3plus/primer3plus.cgi; Supplementary Table 1). PCR conditions were 3 min at 94 °C followed by 30 cycles of 94 °C for 30 s, 60 °C for 30 s and 72 °C for a time period that depends on the predicted product size. PCR amplification was monitored on 1% agarose gel. For APA validation, we used 3′ RACE (Invitrogen) strategy for cDNA synthesis and PCR amplification. cDNA was prepared using adaptor primer and PCR was carried out using an abridged universal (AUAP) reverse primer and gene-specific forward primer (Supplementary Table 1). To confirm differential gene expression in response to PEG treatment, cDNA from control and treated tissues was used in qPCR with SYBR green master 1 mix (Roche) and gene-specific primers (Supplementary Table 1). UBQ was used as an internal control for normalization. Full uncropped images of all gels used to prepare Figs 3, 4, 6 and Supplementary Fig. 7 are shown in Supplementary Fig. 8.

### Cloning and sequencing of novel isoforms

PCR products were excised from the gel and purified using a gel extraction kit (Qiagen). The purified products were cloned onto pGEM-T Easy cloning vector (Promega) and sequenced from both directions using T7 and SP6 primers. Sequences were aligned to the genome using the online version of Spidey (http://www.ncbi.nlm.nih.gov/spidey/) to predict exons and introns. The consensus splicing sites (GT/AG) were manually confirmed and structures of the novel isoforms were predicted.

### Data availability

Iso-Seq alignments have been deposited into the European Nucleotide Archive under accession no. PRJEB13026. The raw reads are available at Zenodo with the identifier http://dx.doi.org/10.5281/zenodo.49944). All other data are available upon request. The TAPIS pipeline is an open source package available from Bitbucket (https://bitbucket.org/comp_bio/tapis) and at our software webpage (http://www.cs.colostate.edu/~asa/software.html).

## Additional information

**How to cite this article:** Abdel-Ghany, S. E. *et al*. A survey of the sorghum transcriptome using single-molecule long reads. *Nat. Commun.* 7:11706 doi: 10.1038/ncomms11706 (2016).

## Supplementary Material

Supplementary Information Supplementary Figures 1 - 8 and Supplementary Table 1

Supplementary Data 1 Alignment of splice isoforms with the genomic sequence.

Supplementary Data 2 Location and distribution of 3' ends of reads within annotated genes.

Supplementary Data 3 List of differentially expressed genes in response to drought treatment as inferred from Iso-Seq

Supplementary Data 4 Novel genes that have a significant tblastx hit with other plant genes.

Supplementary Data 5 A summary of miRNAs identified in Iso-Seq data.

## Figures and Tables

**Table 1:** Alignment statistics of Iso-Seq reads to the reference genome with various error correction methods.

**Method**	**Aligned**	**Junction-filtered**	**Stranded**	**Unique**	**Multi-reads**
LoRDEC	881,052	730,713 (82.94%)	730,593 (82.92%)	700,735 (79.53%)	29,858 (3.39%)
Proovread	880,720	674,346 (76.57%)	674,137 (76.54%)	643,631 (73.08%)	30,506 (3.46%)
TAPIS-ref	845,996	803,041 (94.92%)	801,484 (94.74%)	783,103 (92.57%)	18,381 (2.17%)
TAPIS-LoRDEC	863,773	830,221 (96.12%)	827,862 (95.84%)	802,780 (92.94%)	25,082 (2.90%)
TAPIS-Proovread	865,365	830,846 (96.01%)	828,210 (95.71%)	803,957 (92.90%)	24,253 (2.80%)

LoRDEC, Long-Read DBG Error Correction; TAPIS, Transcriptome Analysis Pipeline for Isoform Sequencing.

For each method, we show the number of reads aligned uniquely and the splice junction filtered reads that could be assigned to a strand. In the proovread and LoRDEC methods, Iso-Seq reads are first corrected using Illumina reads and then aligned to the genome. The TAPIS-ref method iteratively aligns and corrects reads using only the reference genome assembly. In the hybrid approaches (TAPIS-LoRDEC and TAPIS-*proovread*), reads are corrected using both Illumina data and then provided as input to the TAPIS-ref method.
