# Supplementary material for: A survey of the sorghum transcriptome using single-molecule long reads
Source: Nat Commun. 2016 Jun 24;7:11706. doi: 10.1038/ncomms11706 (PMC4931028; doi:10.1038/ncomms11706)
Supplement: Supplementary Information — Supplementary Figures 1 - 8 and Supplementary Table 1 [file ncomms11706-s1.pdf]

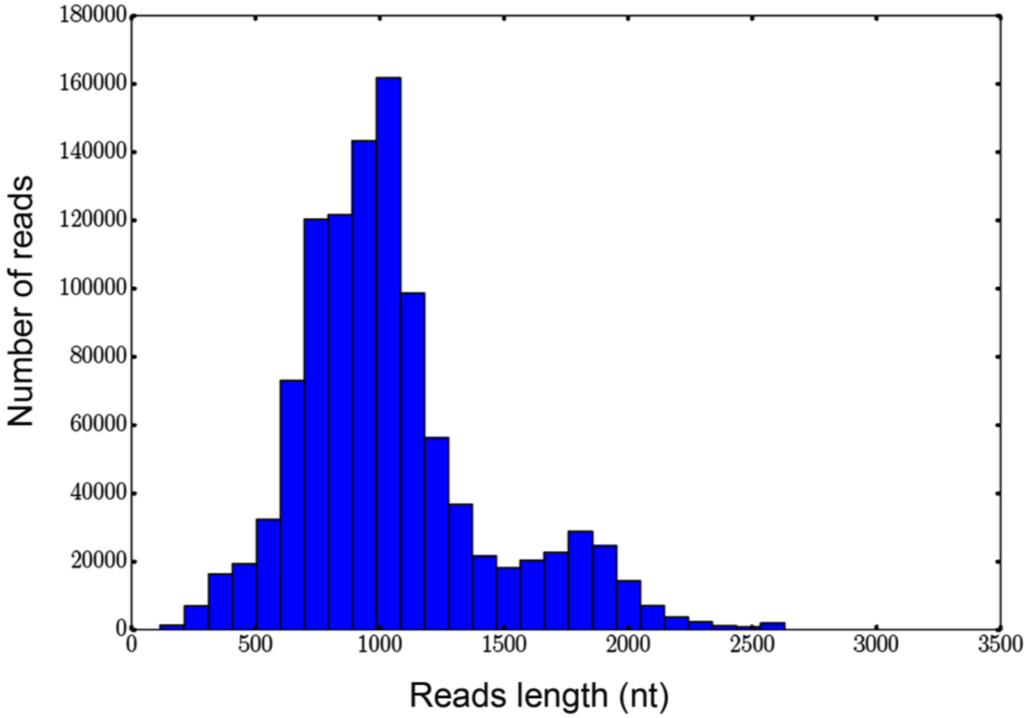

Supplementary Figure 1: Length distribution of Iso-Seq reads

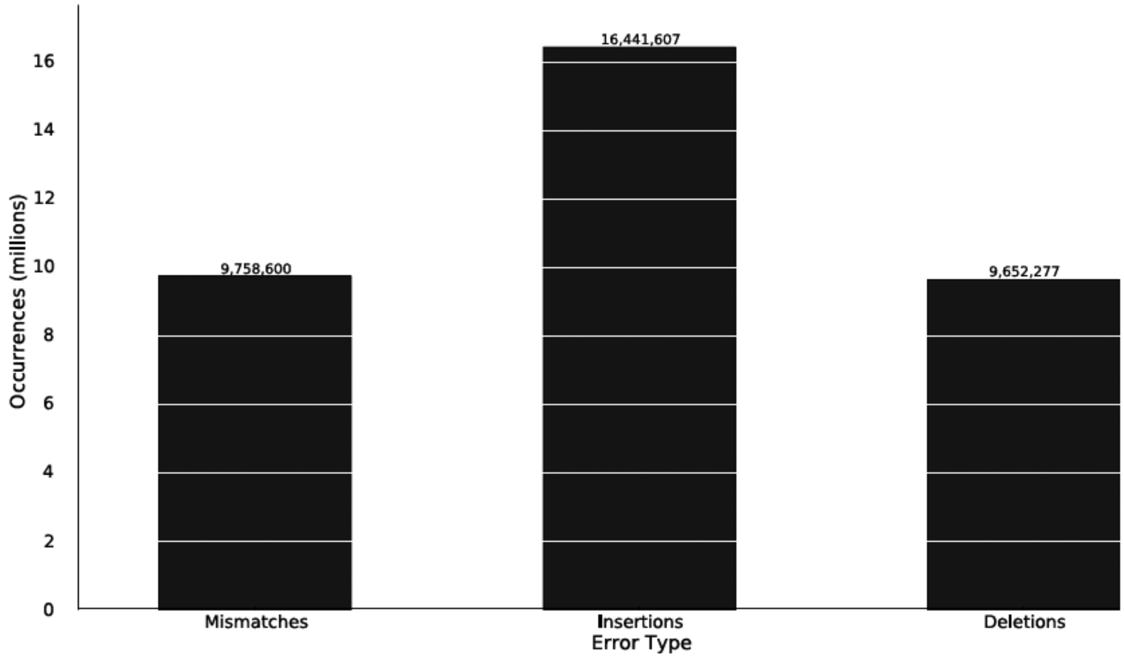

Supplementary Figure 2: Errors in Iso-Seq reads. Distribution of different types of errors in Iso-Seq data.

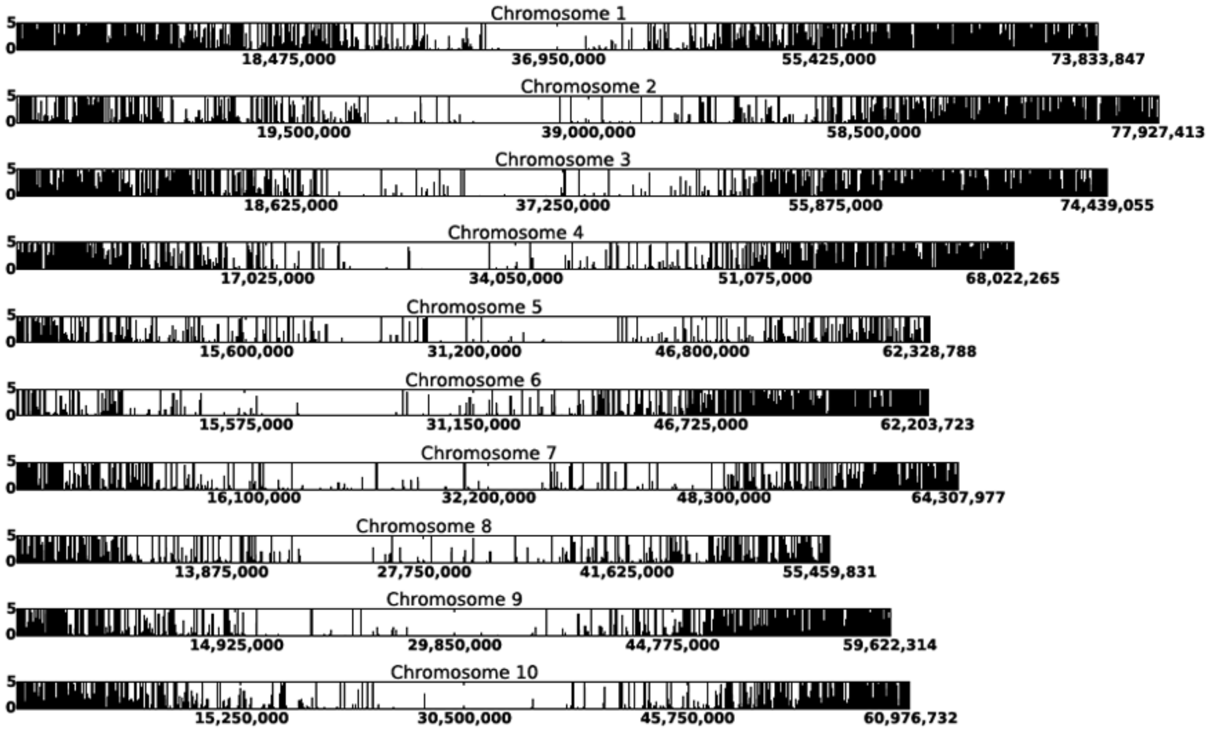

Supplementary Figure 3: Distribution of Iso-Seq reads along sorghum chromosomes

Gene: Sb04g006450
Location: Chr04: 6437981-6440779 forward
Description: Similar to KH domain-containing protein-like

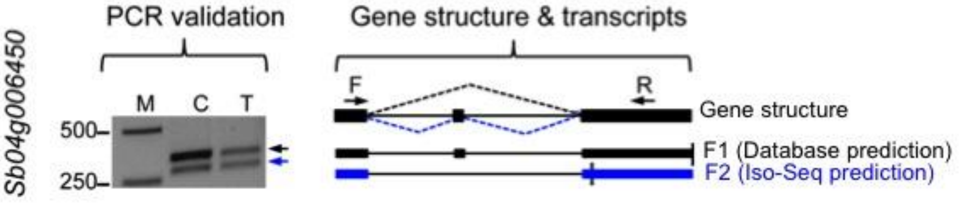

Gene: Sb08g020910
Location: Chr08: 52285286-52288546 forward
Description: Similar to Sterol desaturase family protein, expressed

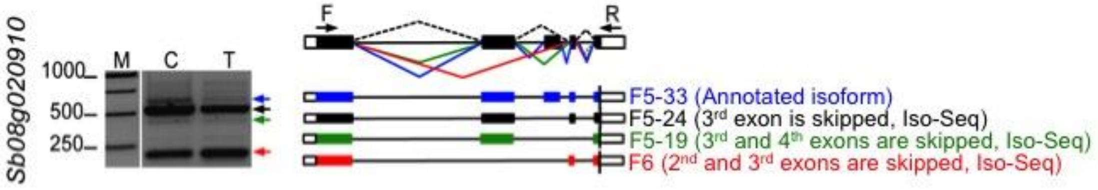

Gene: Sb08g004280
Location: Chr08: 5024397-5027834 forward
Description: Weakly similar to OSJNBb0003B01.20 protein

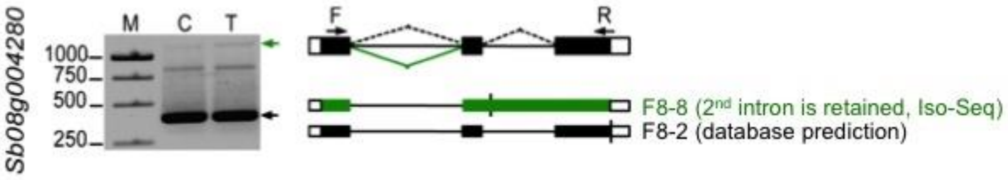

Supplementary Figure 4 (page 1 of 3)

Gene: Sb05g013400
Location: Chr05: 27748219-27752159 forward
Description: Similar to protein family UPF0016 containing protein

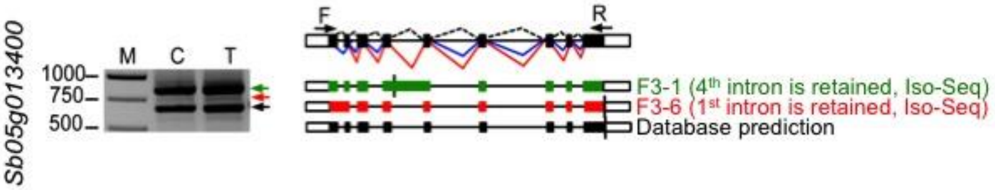

Gene: Sb04g021010
Location: Chr04: 49187968-49192259 forward
Description: Similar to Glycerophosphoryl diester phosphodisterase

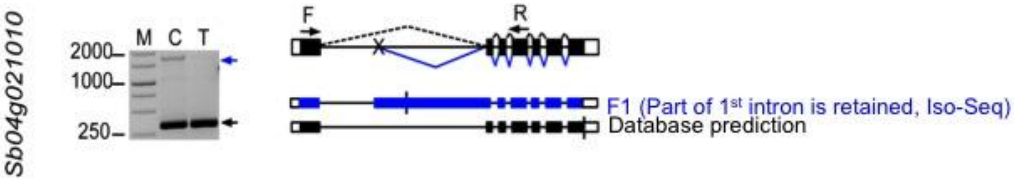

Gene: Sb04g005880
Location: Chr04: 5686493-5689213 forward
Description: similar to Metal-dependent hydrolase-like protein

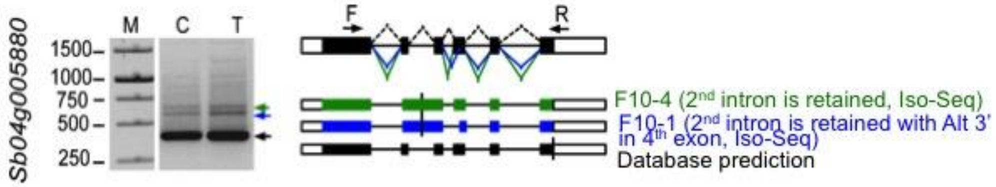

Supplementary Figure 4 (page 2 of 3)

SbmiR159a
>Chr03 Chr03:8124426..8124989 (- strand) class=match length=564

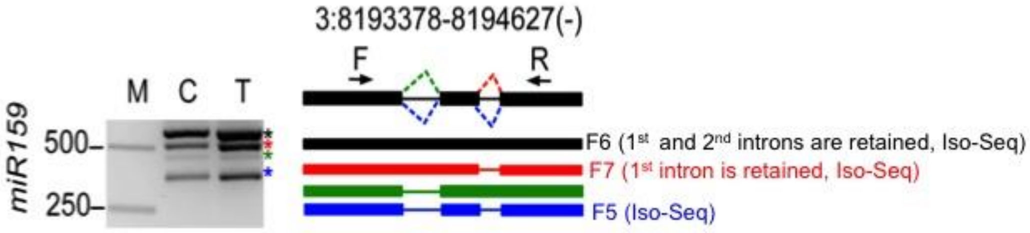

SbmiR166
Chromosome: Sorbi1: 10:59811600: 59812000:1

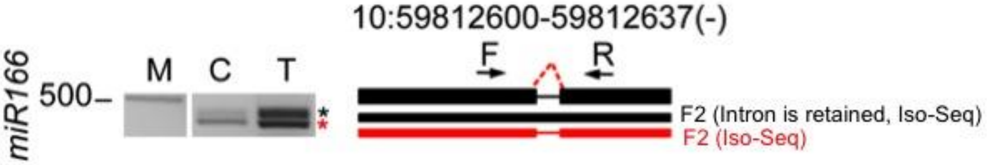

SbmiR444
>Chr04 Chr04:58976044..58981784 (+ strand) class=match length=5741

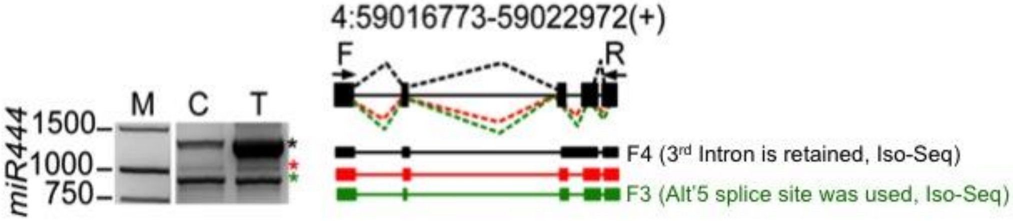

Supplementary Figure 4. PCR amplification of splice isoforms (left) and schematic representation (right) of different splice isoforms. The number adjacent to each isoform indicates the clone that provided evidence for that isoform. Exons are represented by boxes and introns by lines. Dotted lines represent the splicing events. Location of primers used in PCR are indicated with arrows. F, forward primer; R, reverse primer. Colored asterisks next to amplicons correspond to the same colored isoform in the schematic diagram.

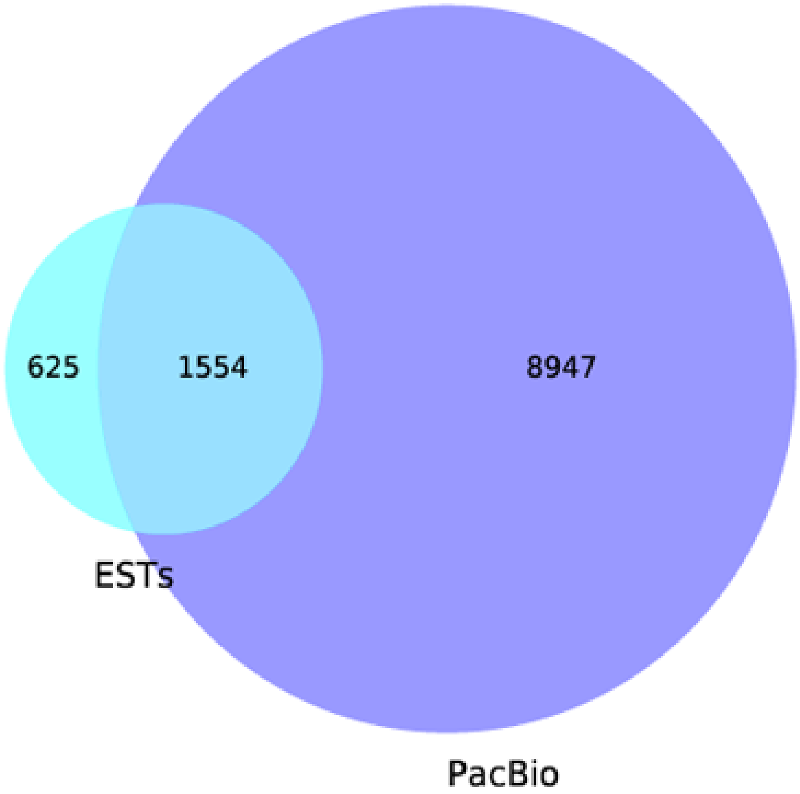

Supplementary Figure 5: Overlap of poly(A) sites in ESTs and Iso-seq. Overlap of poly(A) sites found in EST data with sites found in our pipeline using Iso-Seq data from genes expressed in both datasets. Most sites (about 96%) were detected, indicating our method can provide reliable insight into poly(A) and more specifically, APA, from Iso-Seq reads.

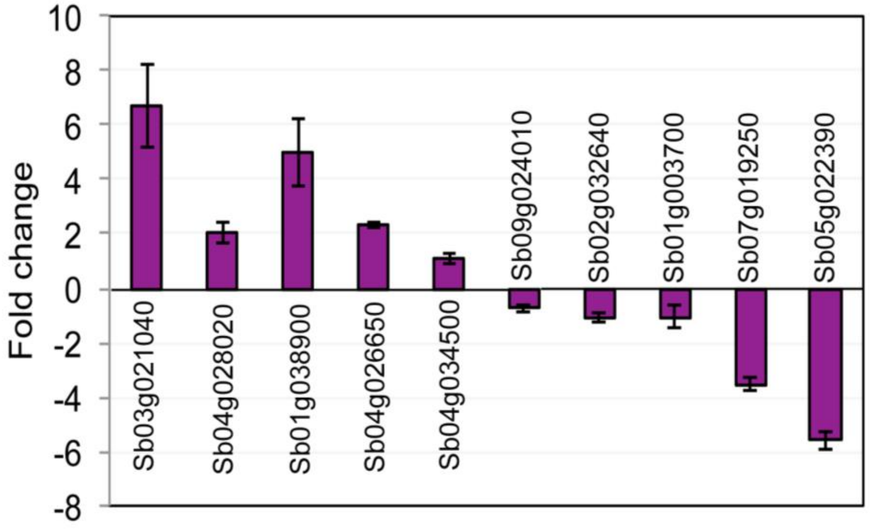

Supplementary Figure 6. Validation of differentially expressed genes. cDNA from control and treated tissues was used in qPCR to quantify gene expression. Expression was normalized to UBQ gene and then to control condition. Upward and downward columns represent induced and repressed genes, respectively, in response to drought treatment. Two biological replicates were used for each sample. Three technical replicates were done for each biological replicate. The mean of three technical replicates of each sample was used as the value of each biological replicate. Minimum and maximum value of expression for each gene is shown.

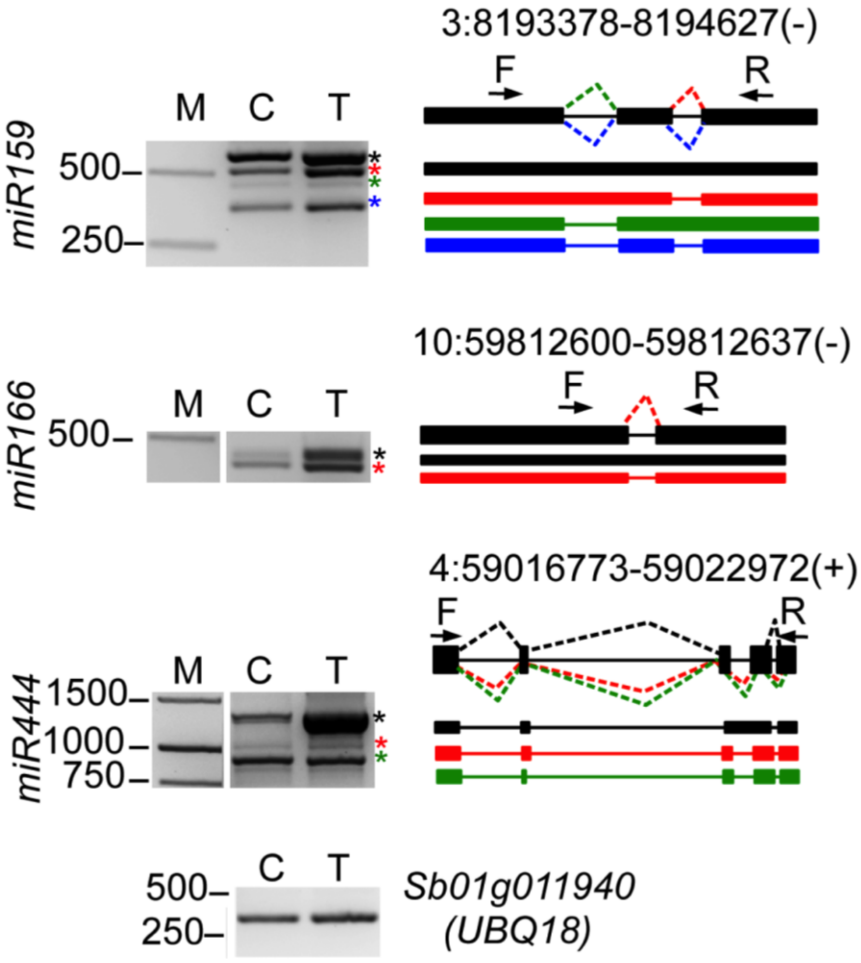

Supplementary Figure 7 : Validation of expression and splicing of miRNAs by PCR. cDNAs prepared from control and treated tissues were used for validation of selected miRNA genes using primers designed to flank the splice junctions. PCR products were purified and sequenced. Exons are represented by filled boxes and introns by lines. Primer locations are represented by arrows. Chromosomal locations are shown above each panel. UBQ was used as an internal control for equal loading.

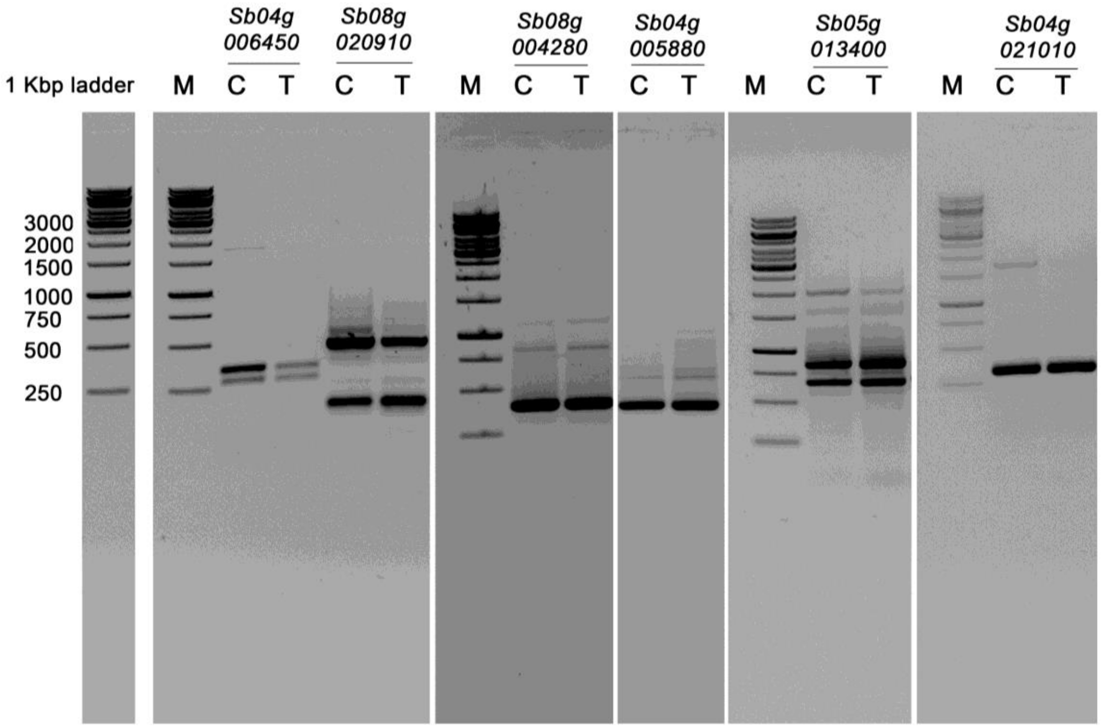

Supplementary Figure 8 (page 1 of 4)

Uncropped images used to prepare Figure 3

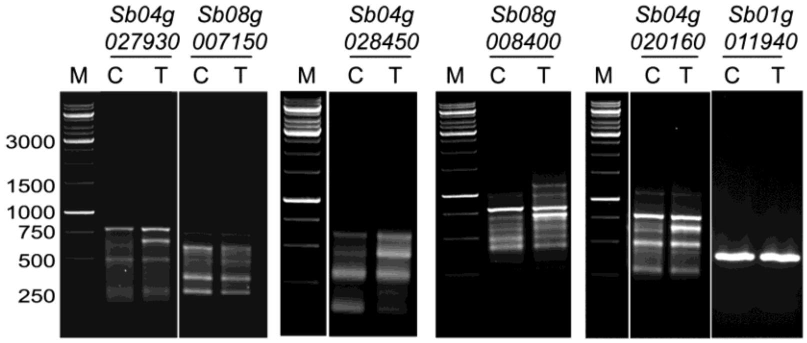

Supplementary Figure 8 (page 2 of 4)

Uncropped images used to prepare Figure 4

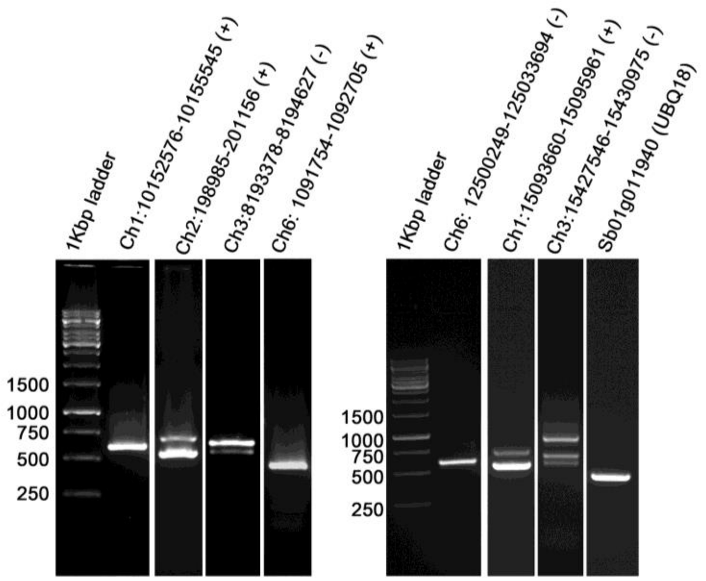

Supplementary Figure 8 (page 3 of 4)

Uncropped images used to prepare Figure 6

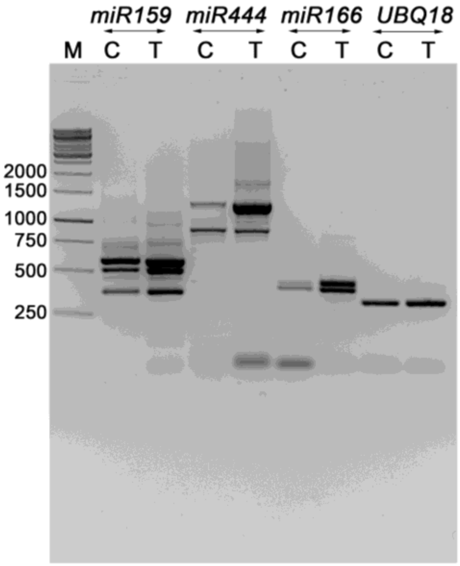

Supplementary Figure 8 (page 4 of 4)

Uncropped images used to prepare supplementary Figure 7

Supplementary Figure 8. Full uncropped images of all gels used to prepare Figures 3, 4, 6 and the supplementary Figure 7

Supplementary Table 1. Sequences of primers used for validation of splicing, alternative polyadenylation sites, predicted miRNAs, differentially expressed and novel genes. ES, exon skipping; IR, intron retention; Alt. 5', alternative donor site; Alt. 3', alternative acceptor site; DE, differential expression; (+) forward strand; (-) reverse strand. Coordinates of the novel and unannotated genes are indicated.

Primer Name	Sequence (5'-3')	Locus	Validation
4G078100-F	ATGGACGGCGGCCGCAAGA	*Sb04g006450*	ES
4G078100-R	AAGTGGCATTTGTCACCCCATT		
8G162700-F	CCACTTATCTACTCCGCTGCA	*Sb08g020910*	ES
8G162700-R	GATGGGTATATGTTCACCGTTCA		
5G111900-F	CTCCTCCTCGCCATCATCGTC	*Sb05g013400*	IR
5G111900-R	TGCTCCGACTGCAACTCCAAT		
4G157300-F	GACTACGTCGAGTTCGACGTCCA	*Sb04g021010*	Alt. 3'
4G157300-R	GTGAGCTCTTCCTCCTGGTAT		
4G070000-F	GACAAGCTCGACCTCGACGT	*Sb04g005880*	IR/Alt. 3'
4G070000-R	CAGTGCAACATATATACTCCAG		
8G050800-F	AGCAGGATGGCAAATACTTCAAC	*Sb08g004280*	IR
8G050800-R	CTTTCTCCTCCTGCACTTGACA		
F9L-F	CATAGTAAATCCATCTTCTACG	*Sb04g020160*	Poly (A)
AdenylateK-F	GATTGACTATTACTCCAAGAAGG	*Sb08g008400*	
PyruvDH-F	GTCCTTGAGATGGATACCTACA	*Sb04g028450*	
GDSL-F	TTGCATCATTCAGGCATCCGG	*Sb08g007150*	
DBP-F	CTGAGCTCAATTAGCTAGGGT	*Sb04g027930*	
miR166-F	GGTGGTGTAGATCTCGGACCA	*Chr10:59812600-*	miRNAs
miR166-R	CCATCATTACACCAATCTGCATC	*59812637 (-)*	
miR444-F	AGACTCGCCGATACGATCCTT	*Chr04:59016773-*	
miR444-R	CACATATATACAGGCAATGCAAAGAG	*59022972 (+)*	
miR159-F	CCAAGATTCAATAGGCACGGT	*Chr03:8193378-*	
miR159-R	CAAACAAGCTTGGCGCAACCAT	*8194627 (-)*	
A-novel-F	TGTCATGTGCAGGGTCACATG	*Chr01:10152576-*	Novel genes
A-novel-R	GACCTCCTAGGTGTAGCTGGT	*10155545(+)*	& long non
B-novel-F	GTACGCTTGGGATAAATTCAG	*Chr02:198985-*	coding
B-novel-R	AAGCCATCATCTCCTATCAGC	*201156(+)*	RNAs
C-novel-F	CAAGCTTGGCGCAACCATGGCT	*Chr03:8193378-*	
C-novel-R	GATTCAATAGGCACGGTCTGC	*8194627(-)*	
D-novel-F	GTGCAGGAAAGTGCATCCTCAA	*Chr06:1091754-*	
D-novel-R	AATGGACAATGAATTGATCTGG	*1092705(+)*	
E-novel-F	GAAGTCTTAGCTTGCTGTCCATC	*Chr06;12500249-*	
E-novel-R	CTCCTATGCAAGCTGCAAAGCTG	*12503694(-)*	
LncRNA1-F	AGGTGCTCATGGTGAAGAGGTG	*Chr01:15093660-*	
LncRNA1-R	ACAGACAATGTATTCCCATTGTA	*15095961(+)*	
LncRNA2-F	TGCCATCCTGTGTAGCTGTGGT	*Chr0:15427546-*	
LncRNA2-F	TACAGCAGACCTTCTGAACTTG	*15430975(-)*	
UBQ18-SQ-F	AGACCTACAAGCTGCAGGTGGA	*Sb01g011940*	Internal

UBQ18-Q-R	AAAGCAACGCCCAGCCGATAA		control
UBQ18-R	GGACAATCAGGAAACCCATCAC		
AP	GGCCACGCGTCGACTAGTAC(T)$_{17}$	*Adaptor primer*	3'RACE
AUAP-R	GGCCACGCGTCGACTAGTAC	*Reverse primer*	
Sb03g021040-QF	CGCCATGTCATATAGCCACT	*Sb03g021040*	
Sb03g021040-QR	ACCATAGCCATTGTGTGGTG		
Sb04g028020-QF	TTGCATTACGTCCATCTTCG	*Sb04g028020*	
Sb04g028020-QR	CCAGAGTCCGGTGTAGAGGT		
Sb01g038900-QF	AGCCGTGTCAGATGAAGTTG	*Sb01g038900*	
Sb01g038900-QR	AGGCTATATCCGCCAAGCTA		
Sb04g026650-QF	GCTTCCAGAGCGCCTACTAC	*Sb04g026650*	
Sb04g026650-QR	GAAGACGGTGTAGACGAGCA		
Sb04g034500-QF	CTGAGGAAGCTGCAGAAGTG	*Sb04g034500*	
Sb04g034500-QR	TGAACCTCTGGTCAGTGCTC		
Sb09g024010-QF	TTGAGCCACTGCCTATCAAG	*Sb09g024010*	Validation of differentially expressed genes by RT-qPCR
Sb09g024010-QR	GAACTGGTTGCCAGAAGACA		
Sb02g032640-QF	GGAACTAGGCCATGCTATCC	*Sb02g032640*	
Sb02g032640-QR	AGGACACCACCATCAACAAA		
Sb01g003700-QF	TTGGATGGTCTTCAACCAGA	*Sb01g003700*	
Sb01g003700-QR	TAGGCGAGTCGGATTTCTCT		
Sb07g019250-QF	ACAGAGCTGCAATTGTTTGG	*Sb07g019250*	
Sb07g019250-QR	CCCTCCCTACCACTATTCCA		
Sb05g022390-QF	ATCGAGTGGAGCATCTGTTG	*Sb05g022390*	
Sb05g022390-QR	GAAATGCGACTTTCAGGGTT		
